# Edge_MVSFormer: Edge-Aware Multi-View Stereo Plant Reconstruction Based on Transformer Networks

**DOI:** 10.3390/s25072177

**Published:** 2025-03-29

**Authors:** Yang Cheng, Zhen Liu, Gongpu Lan, Jingjiang Xu, Ren Chen, Yanping Huang

**Affiliations:** Guangdong-Hong Kong-Macao Joint Laboratory for Intelligent Micro-Nano Optoelectronic Technology, School of Physics and Optoelectronic Engineering, Foshan University, Foshan 528000, China; 2112205029@stu.fosu.edu.cn (Y.C.); 20220240309@stu.fosu.edu.cn (Z.L.); langongpu@fosu.edu.cn (G.L.); xujingjiang@fosu.edu.cn (J.X.); chenren@fosu.edu.cn (R.C.)

**Keywords:** plant 3D reconstruction, multi-view stereo, depth image, point cloud, edge regions, edge detection, edge-aware loss function, transformer, reconstruction error

## Abstract

With the rapid advancements in computer vision and deep learning, multi-view stereo (MVS) based on conventional RGB cameras has emerged as a promising and cost-effective tool for botanical research. However, existing methods often struggle to capture the intricate textures and fine edges of plants, resulting in suboptimal 3D reconstruction accuracy. To overcome this challenge, we proposed Edge_MVSFormer on the basis of TransMVSNet, which particularly focuses on enhancing the accuracy of plant leaf edge reconstruction. This model integrates an edge detection algorithm to augment edge information as input to the network and introduces an edge-aware loss function to focus the network’s attention on a more accurate reconstruction of edge regions, where depth estimation errors are obviously more significant. Edge_MVSFormer was pre-trained on two public MVS datasets and fine-tuned with our private data of 10 model plants collected for this study. Experimental results on 10 test model plants demonstrated that for depth images, the proposed algorithm reduces the edge error and overall reconstruction error by 2.20 ± 0.36 mm and 0.46 ± 0.07 mm, respectively. For point clouds, the edge and overall reconstruction errors were reduced by 0.13 ± 0.02 mm and 0.05 ± 0.02 mm, respectively. This study underscores the critical role of edge information in the precise reconstruction of plant MVS data.

## 1. Introduction

In agricultural and plant biology research, analyzing phenotypic parameters is crucial for evaluating plant growth, development, and their interactions with genetic and environmental factors [1]. Traditionally, these analyses have been labor-intensive and sometimes destructive. However, with technical advancements, three-dimensional (3D) modeling has emerged as an important approach [2,3]. These innovative techniques rely on imaging and computational analysis to accurately reconstruct 3D plant structure and measure various phenotypic traits of the plant [4,5], which are essential for assessing its health, yield potential, and responses to environmental stresses [6]. In fact, 3D reconstruction techniques are also widely used in the manufacturing industry; for example, He et al. [7] effectively improved the manufacturing efficiency and automation level in the field of thick plate arc welding by using a laser vision system to inspect the weld seam and torch, extract variable weld contours, and optimize the weld position through the Analytic Hierarchy Process (AHP).

Recent advances in 3D imaging technologies have significantly improved precision agriculture and plant science, enabling detailed 3D reconstruction of plant structures and growth parameters [4,8]. Depending on whether the acquisition device emits measurement signals, 3D reconstruction can be categorized into active and passive methods [9]. Active 3D reconstruction includes techniques such as laser scanning [10,11], structured light methods [12], and Kinect-based approaches [13,14]. For instance, Aanæs et al. [15] used structured light to generate a diverse multi-view stereo (MVS) dataset, while Teng et al. [13] and Hu et al. [14] employed Azure Kinect cameras to capture the structure of oilseed rape and leafy vegetables from multiple angles, generating high-quality point clouds for plant phenotyping. However, the accuracy of these methods depends on specific measurement instruments, which are often expensive and suitable only for specific scenarios, making them less cost-effective for widespread use. Passive 3D reconstruction is classified into traditional and learning-based methods based on data processing and modeling approaches [9]. Traditional geometry-based MVS matching algorithms, such as Gipuma [16] and COLMAP [17], rely on similarity search across views. However, these methods struggle with weakly textured regions and reflective surfaces, which can negatively impact reconstruction accuracy.

Learning-based methods implicitly encode information such as illumination and reflection into image features during the reconstruction process. This rich semantic information helps improve object reconstruction in scenes with weak textures and reflective surfaces. The initial MVS matching network, which is based on end-to-end learning, adopts voxel-based reconstruction. In this approach, the 3D scene is divided into voxel grids, and the extracted image features are back-projected into 3D space based on camera parameters. A local smoothing term is applied using 3D convolution to the resulting 3D scene raster, and the final output is a voxel-occupied representation of the scene. Another approach uses plane sweeping [18], which operates on the principle that each depth value is associated with a projection of the source image onto a parallel plane in front of a reference camera. A confidence level for each depth is inferred based on parallax, and a “winner-takes-all” strategy is used to select the most reliable depth. Yao et al. [19] proposed MVSNet, an MVS matching network based on the plane sweeping algorithm, which integrates the parallax regression strategy commonly used in stereo matching tasks. However, the need to select depth intervals and perform 3D convolutions can be memory-intensive. To address this, Gu et al. [20] proposed CasMVSNet, which uses a coarse-to-fine strategy to progressively reduce the depth range, ultimately producing high-resolution depth maps. Zhang et al. [21] introduced GeoMVSNet, a two-branch fusion method combining coarse depth maps enriched with geometric priors and features from classical feature pyramid networks (FPNs). By incorporating enhanced 2D regularization, this method avoids the computational burden of 3D convolutions while maintaining high-quality depth reconstruction. Building on the success of transformer models in natural language processing [22], Ding et al. [23] introduced a transformer-based architecture for 3D reconstruction, known as TransMVSNet. This model improves feature matching by combining self-attention and cross-attention mechanisms, allowing it to aggregate long-range contextual information within and between images, significantly enhancing matching accuracy. He et al. [24] proposed an innovative multi-view stereo (MVS) network called MVSFormer, which enhances feature learning by integrating pre-trained vision transformers, significantly improving the accuracy and efficiency of 3D reconstruction. These advanced algorithms perform excellently on several publicly available 3D reconstruction datasets; however, their accuracy still faces challenges when dealing with complex structures such as plant leaf edges. The edge regions of plant leaves usually have complex textures and geometries, leading to more significant depth estimation errors, which limits the effectiveness of its application in plant 3D reconstruction. In addition, existing methods often neglect the importance of edge information during the reconstruction process, which is crucial for improving the reconstruction accuracy of plant leaf edges.

In this study, we proposed the Edge_MVSFormer network model based on the TransMVSNet for reconstructing 3D plant models from multi-view RGB images, focusing on more accurate depth map estimation and subsequent point cloud generation. Using the point cloud obtained from the Freescan X3 laser scanner as the ground truth and TransMVSNet as a benchmark, the results show that our method significantly improves the accuracy of leaf edge depth estimation and point cloud generation, with a general enhancement of the overall plant depth image and point cloud reconstruction.

## 2. Materials and Methods

**Figure 1:**
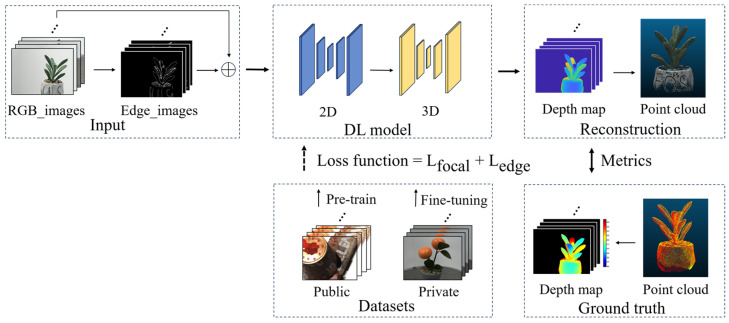
The study’s overall flowchart illustrates the following processes: multi-view images of a plant and its ground truth point cloud are obtained using an RGB camera and a Freescan X3 laser scanner, respectively. RGB images and edge information are concatenated as network inputs, and the output was a single-view depth image, multiples of which are fused to obtain a complete point cloud of the whole scene. The ground truth depth image is calculated based on the geometric relationship between the ground truth point cloud and the specific view angle, which is used for network fine-tuning and depth map evaluation, respectively. Finally, the quality of the reconstructed depth map and the reconstructed point cloud are evaluated using evaluation metrics.

Figure 1 illustrates the overall processing steps of the applied method in this paper, which leverages a deep learning model to generate high-quality depth images from adjacent multi-view inputs and subsequently fuses them to construct a complete point cloud. RGB images, along with corresponding edge information, are concatenated and used as inputs. To enhance depth estimation accuracy, particularly along edges, the model incorporates both focal loss and edge-aware loss, effectively reducing depth estimation errors and improving the quality of the reconstructed point cloud. The deep learning model is pre-trained on two public datasets and fine-tuned with a private dataset specifically created for this study. The method’s performance is evaluated based on the reconstructed depth images and point clouds separately.

### 2.1. Data Preparation

#### 2.1.1. Public Dataset

In this study, the DTU and BlendedMVS datasets were adopted for the model pre-training [23]. DTU [15] consists of 128 scenes captured in a controlled laboratory environment, each under 7 different lighting conditions and viewed from 49 or 64 camera positions. Its ground truth point cloud model was obtained with a structured light scanner. The BlendedMVS [25] contains 113 scenes, including objects ranging from buildings and streetscapes to sculptures and small objects. Each scene contains between 20 and 1000 input images, with the entire dataset containing more than 17,000 images in total. More details related to these two datasets can be found in the given references.

#### 2.1.2. Private Dataset and Preparation

**Figure 2:**
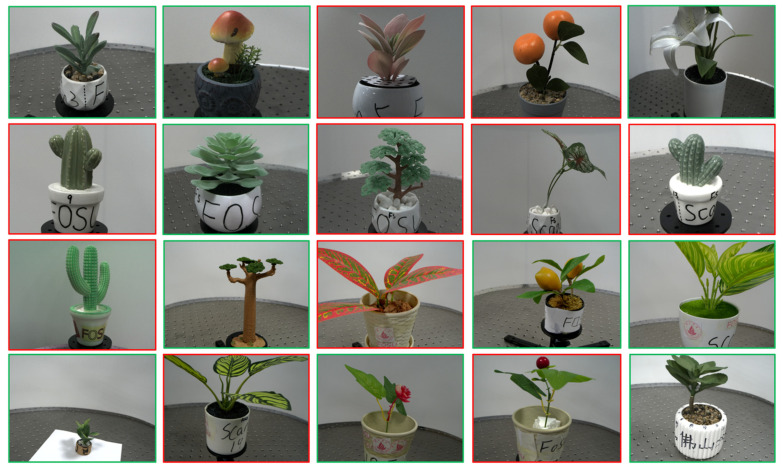
Pictures of 20 plant models. Red borders indicate plants used for DL model fine-tuning, and green is used for testing.

**Figure 3:**
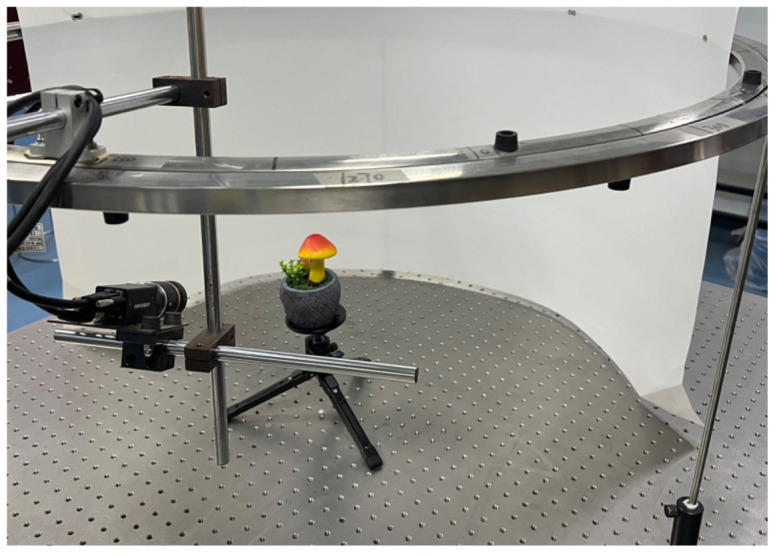
Image acquisition system custom-designed in this study. The height and tilt of the camera can be freely adjusted by means of connecting rods to achieve a complete image acquisition at 360° of view.

We selected 20 model plants, including succulents, lilies, begonias, and cacti, as shown in Figure 2, to construct our private dataset. These plants were chosen for their diverse structures, which range from thin objects like leaves and flowers to more spherical structures like fruits. Image acquisition and ground truth point cloud generation were carried out indoors. A custom-designed image acquisition system was developed on an optical platform to collect multi-view plant images, as shown in Figure 3. The system primarily consists of a dual-loop slide rail, a camera, and a computer. The slide rail, with a diameter of 1 m, features a fixed outer loop and a freely movable inner loop. An industrial face array camera (model MV-CA060-10GC, Hikvision Inc., Hangzhou, China) is mounted on the inner loop and connected to a PC running Windows 10 with an Intel Core i5 processor. During data collection, each plant is positioned at the center of the slide rail, and images are captured at 15° intervals from two different heights, yielding 48 multi-view images per plant. In total, 960 images were obtained under normal lighting conditions for the 20 plant specimens. Additionally, to evaluate the generalization ability of the proposed method, we captured multi-view images of the first 10 plant specimens shown in Figure 2 under both red and green lighting conditions. For each lighting condition, 48 images were captured for each plant, resulting in a total of 960 images—480 under red light and 480 under green light.

**Figure 4:**
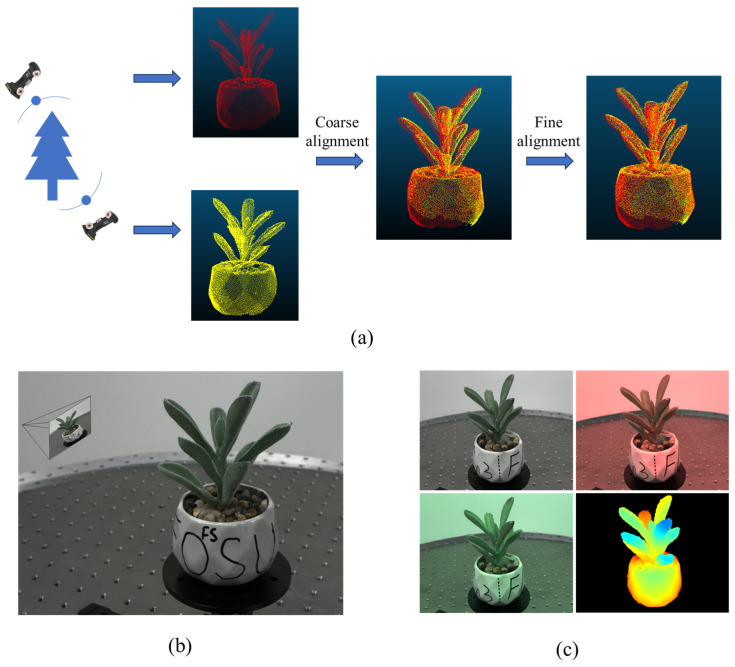
(**a**) Alignment process of two viewpoint point clouds from the laser scanner for a typical sample plant, (**b**) schematic of a viewpoint image relative to a plant model, (**c**) the corresponding viewpoint RGB images under different lighting conditions and their ground truth depth map for the scene shown in (**b**).

In this study, a Freescan X3 handheld laser scanner (Beijing Tianyuan 3D Technology Co., Beijing, China) was used to acquire ground truth point clouds of plant models. This scanner employs laser line scanning technology with a measurement accuracy of up to 0.03 mm. The procedure includes connecting the Freescan X3 to a computer, positioning the plant model in front of a calibration plate, and smoothly moving the scanner around the object. The scan data are then reconstructed using the manufacturer’s proprietary software. To ensure the completeness of the scanned point cloud, the plant was scanned from at least two significantly different angles (with a 180° interval for the two angles; if scanning from three angles, the interval between each view is about 120°). The point clouds obtained from multiple scans were first coarsely aligned and then finely aligned to achieve a complete point cloud structure. Coarse alignment involves a reasonable initial transformation of the two point clouds without focusing on accuracy, while fine alignment is performed by iteratively finding the closest point pairs using the Iterative Closest Point (ICP) algorithm [26]. The alignment process for fusing two point clouds into one is briefly illustrated in Figure 4a.

The ground truth depth image for each RGB image was generated using the ground truth point cloud obtained from the laser scanner along with the camera parameters of each view angle. Specifically, the COLMAP algorithm [17] was employed to reconstruct the multi-view images in 3D, producing the 3D point cloud model of the plant along with the camera parameters for each view. The 3D point cloud from COLMAP was then coarsely and finely aligned with the point cloud from the laser scanner to bring both datasets into the same world coordinate system. Finally, using the geometric relationship between the ground truth point cloud and the camera parameters of each view, the ground truth depth map was computed. A typical RGB image of a succulent plant from a specific viewpoint and its corresponding depth map are shown in Figure 4b,c.

### 2.2. 3D Plant Reconstruction Method

#### 2.2.1. Network Model

**Figure 5:**
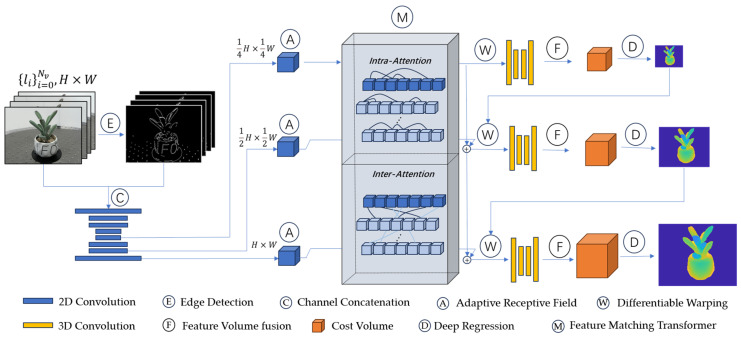
Edge_MVSFormer architecture. Edge_MVSFormer applies the Canny edge detection algorithm to obtain the contour information in the RGB image, concatenates it with the RGB image channels to form a four-channel input, and extracts the multi-scale image features by using the feature pyramid network. Before handing these features to the converter, the Adaptive Receptive Field (ARF) module is used to refine the local feature extraction and ensure the image quality. In order to take full advantage of the global contextual information within and between the reference image and the source image, the Feature Matching Transformer (FMT) is employed to perform internal correlation and mutual correlation. Feature paths of different resolutions at each stage are connected; a variable warping technique is applied to align all source views with the reference view, which are fused to the reference view cost volume using 3D convolution, and a depth image is obtained after depth regression.

The overall architecture of Edge_MVSFormer is illustrated in Figure 5. In this experiment, an end-to-end approach is used to predict the depth map of the reference image given a reference image and $N_{\nu }$ source images with their internal and external parameters known.

#### 2.2.2. Edge Detection

**Figure 6:**
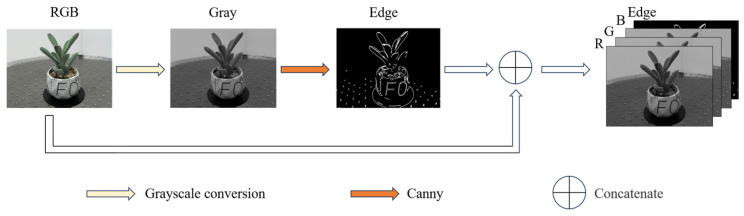
Edge detection and channel concatenation for RGB images.

Edge detection extracts contours and structural details from an image, while raw RGB images primarily provide rich color information. By combining these two types of data, a more comprehensive representation of the image is achieved. In this study, edge detection is performed using the Canny operator, which computes the image gradient in both the x and y directions to generate a single-channel edge map. This edge map is then concatenated with the original RGB image along the channel dimension, resulting in a 4-channel image (R, G, B, and Edge), as shown in Figure 6. The high and low thresholds were experimentally set as 150 and 50, respectively, in Canny edge extraction, which balances edge preservation and noise suppression.

#### 2.2.3. Loss Function

Most of the previous similar studies used an *L*_1_-based depth regression loss function, which monitors the absolute distance between the predicted and ground truth images. Due to the focus on depth estimation of the edge portion of the leaf, the pixel-based classification model is more suitable for our task, where the focal loss at each stage is defined as follows:

(1) $$L_{\mathrm{focal}}=\frac{1}{N}\sum_{i=1}^{N}-{\left(1-P_{i}^{\tilde{d}}\right)}^{\gamma }\log\left(P_{i}^{\tilde{d}}\right)$$

where *N* represents the number of valid pixels, *γ* is the focusing parameter that controls the rate of weight reduction for easy samples (*γ* set as 2 in this study), and $P_{i}^{d}$ denotes the predicted probability of the depth hypothesis *d* at pixel *i*. The depth hypothesis *d* ranges from $d_{\min}$ to $d_{\max}$ and is calculated by the formula $d=d_{\min}+∆d*k$, where *k* represents the division of the depth interval into *k* parts. $\tilde{d}$ is the depth hypothesis closest to the ground truth depth.

Moreover, the focus of this paper is on enhanced edge depth estimation. We use the edge-aware loss function to compute the ground truth depth image and the predicted depth image of the leaf. The edge-aware loss function is defined as follows:

(2) $$L_{\mathrm{edge}}=\frac{1}{M}\sum_{j=1}^{M}E_{\mathrm{gt},j}\times \left|G_{\mathrm{gt},j}-G_{\mathrm{pre},j}\right|$$

where $E_{\mathrm{gt},j}$ is the edge mask obtained using the Canny edge detection operator for the ground truth depth image, *M* is the number of the corresponding set of pixels in the edge mask, and $G_{\mathrm{gt},j}$ and $G_{\mathrm{pre},j}$ represent the gradient magnitude of the ground truth depth image and the predicted ground truth depth image, respectively.

The overall loss function is a weighted sum of $L_{\mathrm{focal}}$ and $L_{\mathrm{edge}}$ at each of a total of three stages, which is defined as follows:

(3) $$L_{\mathrm{total}}=\sum_{s=1}^{3}\left(\lambda_{1}^{s}\times L_{\mathrm{focal}}^{s}+\lambda_{2}^{s}\times L_{\mathrm{edge}}^{s}\right)$$

where $\lambda_{1}^{s}$ and $\lambda_{2}^{s}$ are experimentally set as 0.9 and 0.1 for each stage in this experiment.

#### 2.2.4. Point Cloud Generation

After obtaining depth images from each viewpoint, the final step is to fuse these depth images within the same scene. Due to the complexity of the plant structure, the error of the background depth estimation can seriously affect the fusion process; therefore, the acquired depth images are first filtered using the main part of the plant in the image as the valid pixel points to remove those unreliable pixel points in the background. Then, the depth map is reprojected for geometrically consistent filtering according to Zhang et al. [27]. Specifically, for a pixel *t_r_* in the reference depth map *D_r_*, its reprojected pixel *t_source_* and depth *d_source_* of its corresponding pixel point can be obtained from each source view. We consider the estimated *D_r_* (*t_r_*) as geometrically consistent for this source view if and only if $∥t_{r}-t_{\mathrm{source}}∥\;<1$ and $\frac{\left|D_{r}\left(t_{r}\right)-d_{\mathrm{source}}\right|}{\max\left(D_{r}\left(t_{r}\right),d_{\mathrm{source}}\right)}<1\%$. Geometric averaging fuses reprojected depths from geometrically consistent source depth maps that are averaged to reduce the noise in depth estimation.

### 2.3. Experimental Validation

#### 2.3.1. Experiment and Algorithm Implementation

In this study, we conducted six controlled experiments on the following four network models: CasMVSNet, GeoMVSNet, TransMVSNet, and Edge_MVSFormer. Each model was initially trained on the DTU and BlendedMVS datasets. Among them, TransMVSNet and Edge_MVSFormer were further fine-tuned using 10 normal-light plant samples from our private dataset, resulting in the fine-tuned versions TransMVSNet_t and Edge_MVSFormer_t, respectively. Additionally, we assessed the generalization ability of all four models in 10 plant scenes under challenging lighting conditions (red and green light).

All experiments were conducted on an Autodl remote server (autodl.com) equipped with an Intel Platinum 8358P CPU, 80 GB of RAM, and an NVIDIA A40 48 GB GPU, running Ubuntu with Python 3.6 and PyTorch 1.6.0. For the DTU dataset, the number of input images was set to Nv = 5, with an image resolution of 512 × 640. Training was conducted for 10 epochs with a batch size of 4. A three-stage regularization strategy, progressing from coarse to fine, was applied, with depth assumptions ranging from 425 mm to 935 mm. The number of depth assumptions for each stage was 48, 32, and 8, respectively. For the BlendedMVS dataset, the number of input images was Nv = 4, the resolution was 576 × 768, the training epochs were 10, and the batch size was 4. The same depth assumptions (48, 32, and 8) were used for each stage. Fine-tuning was performed on our private plant dataset, with an image resolution of 864 × 1152, 10 training epochs, and a batch size of 1. The number of input images was Nv = 4, and the same depth hypotheses were used as in the previous datasets. For all training datasets, the initial learning rate was set to 0.001 for all models, decreasing by a factor of 0.5 after 6 and 8 epochs, respectively [23]. Evaluation was performed on a test set of our private plant dataset, with Nv = 4 and an input image size of 864 × 1152. We assessed the reconstruction accuracy of both the reconstructed depth maps and the fused 3D point clouds.

#### 2.3.2. Evaluation Metrics

##### Evaluation of Reconstructed Depth Images

Ground truth depth images of multi-view images were obtained using the method shown in Figure 4, and the accuracy of the estimated depth maps was assessed by calculating the MAE_Depth between the ground truth depth map and the predicted depth map of the valid pixels, which correspond to regions containing only the plant subject, as follows:

(4) $$\mathrm{MAE}\_\mathrm{Depth}=\frac{1}{\mathrm{Nz}}\sum_{i=1}^{\mathrm{Nz}}\left|D_{\mathrm{gt}}\left(i\right)-D_{\mathrm{pre}}\left(i\right)\right|$$

where *Nz* is the number of valid pixel points in the depth image, $D_{\mathrm{gt}}\left(i\right)$ is the depth of field at pixel point *i* in the ground truth depth image, and $D_{\mathrm{pre}}\left(i\right)$ is the depth of field at pixel point *i* in the test depth image. The proportion of pixel points with errors below a predetermined threshold is also calculated and referred to as “EPE_n”, where n is the threshold value in a unit of millimeters [25].

**Figure 7:**
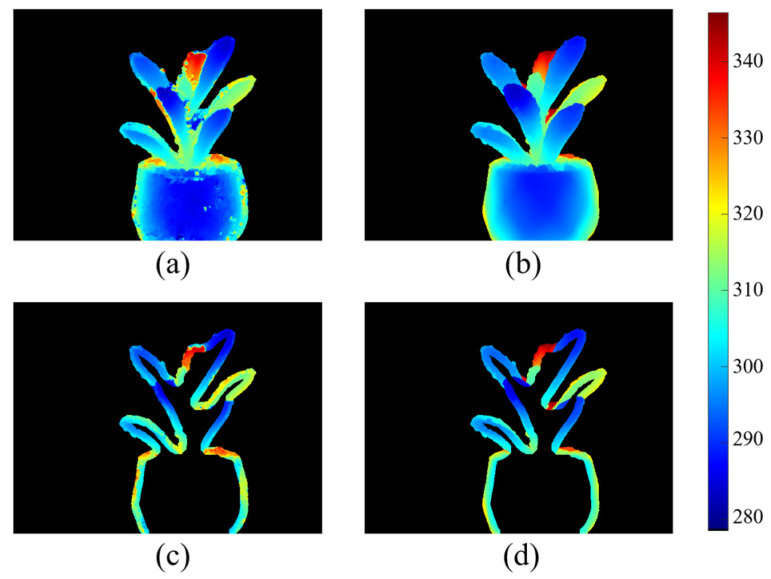
Edge region error calculation for depth images. (**a**) is the test depth image obtained using our method; (**b**) is the ground truth depth map; (**c**,**d**) are the edge regions of (**a**,**b**), respectively, with a width of 40 pixels; and MAE_Depth is the mean absolute depth error between (**c**,**d**). The color bars represent an absolute depth in millimeters.

To evaluate the impact of edge channels and edge-aware functions on the depth estimation of leaf edges in depth images, we assess the ring-shaped precision of the leaf edges in the depth images. As shown in Figure 7, MAE_Depth is computed for pixels within an annular region extending 40 pixels inward from the leaf edge.

To estimate the uncertainty of the results, we evaluated all methods using ten independent plant test samples. For each sample, we calculated the MAE_depth for valid pixels and edge regions in the depth image. The standard deviation (STD) across these ten samples was then computed to capture the variability between different plant instances, providing a measure of uncertainty. This approach effectively reflects the variation in the model’s performance across different samples.

##### Evaluation of Reconstructed Point Clouds

The performance of the reconstructed point cloud is measured using the mean absolute error distance (MAE_Distance) between the reconstructed point cloud and the ground truth point cloud. To evaluate the performance of the reconstructed point cloud as a whole, the average of the distances from each point in the reconstructed point cloud to the nearest point in the ground truth point cloud was calculated as MAE_Distance_1, and the average of the distances from each point in the ground truth point cloud to the nearest point in the reconstructed point cloud was calculated as MAE_Distance_2, respectively. Then the average of the two was used as the final MAE_Distance.

(5) $$\mathrm{MAE}\_\mathrm{Distance}\_1=\frac{\sum_{i=1}^{N}\mathrm{distance}\left(p_{i},p_{i,\mathrm{gt}}\right)}{N}$$

where *N* is the number of points in the reconstructed point cloud, $p_{i}$ is a point in the reconstructed point cloud, $p_{i,\mathrm{gt}}$ represents the point in the ground truth point cloud that has the minimum distance from $p_{i}$.

(6) $$\mathrm{MAE}\_\mathrm{Distance}\_2=\frac{\sum_{j=1}^{M}\mathrm{distance}\left(q_{j},q_{j,\mathrm{rec}}\right)}{M}$$

where *M* is the number of points in the ground truth point cloud, $q_{j}$ is the *j*-th point in the ground truth point cloud, and $q_{j,\mathrm{rec}}$ represents the point in the reconstructed point cloud that has the minimum distance from $q_{j}$.

(7) $$\mathrm{MAE}\_\mathrm{Distance}=\frac{\mathrm{MAE}\_\mathrm{Distance}\_1+\mathrm{MAE}\_\mathrm{Distance}\_2}{2}$$

A distance threshold is also set to evaluate an average probability that points in the reconstructed point cloud are classified to be “correct”. To avoid focusing solely on increasing algorithmic accuracy and retaining only the high-accuracy points while ignoring the completeness of the reconstructed scene, a pair of trade-off metrics were used to jointly evaluate the point cloud reconstruction performance.

(8) $$\mathrm{Acc}=\frac{\sum_{i=1}^{N}I\left(\mathrm{distance}\left(p_{i},p_{i,\mathrm{gt}}\right)<D_{\mathrm{th}}\right)}{N}\times 100\%$$

where $I\left(*\right)$ represents an indicator function, its value is 1 when the condition is true; otherwise, it is 0, and $D_{\mathrm{th}}$ = 0.4 mm. The definitions of $p_{i},p_{i,\mathrm{gt}}$ are the same as in Equation (5).

(9) $$\mathrm{Comp}=\frac{\sum_{j=1}^{M}I\left(\mathrm{distance}\left(q_{j},q_{j,\mathrm{rec}}\right)<D_{\mathrm{th}}\right)}{M}\times 100\%$$

(10) $$\mathrm{OP}=\frac{\mathrm{Acc}+\mathrm{Comp}}{2}$$

where $I\left(*\right)$ is the same function as defined above, the definitions of $q_{j},q_{j,\mathrm{rec}}$ are the same as in Equation (6), and OP is the overall performance.

**Figure 8:**
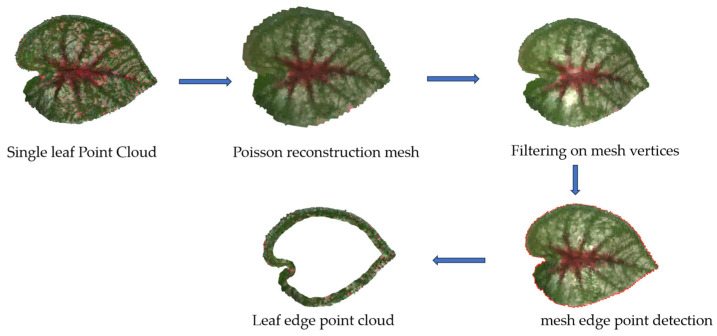
Leaf edge point cloud segmentation process. Poisson reconstruction is applied to convert the leaf point cloud into a smooth mesh. Vertex filtering is then performed on the mesh to address the over-reconstruction issue introduced by Poisson reconstruction. Edge points are detected on the filtered mesh, and a spatial distance threshold of 4 mm is set to segment the reconstructed leaf point cloud.

The leaf edges of the ground truth point cloud and the reconstructed point cloud were segmented using a method shown in Figure 8. The reconstructed point cloud of the leaf edge region was also evaluated using MAE_Distance and OP, which is the same as the whole point cloud evaluation part. Similarly, we calculated the MAE_distance for the reconstructed overall point cloud and leaf edge point cloud of the ten test samples and computed the corresponding STD to reflect the variability in the results.

##### Evaluation of Phenotypic Parameters

**Figure 9:**
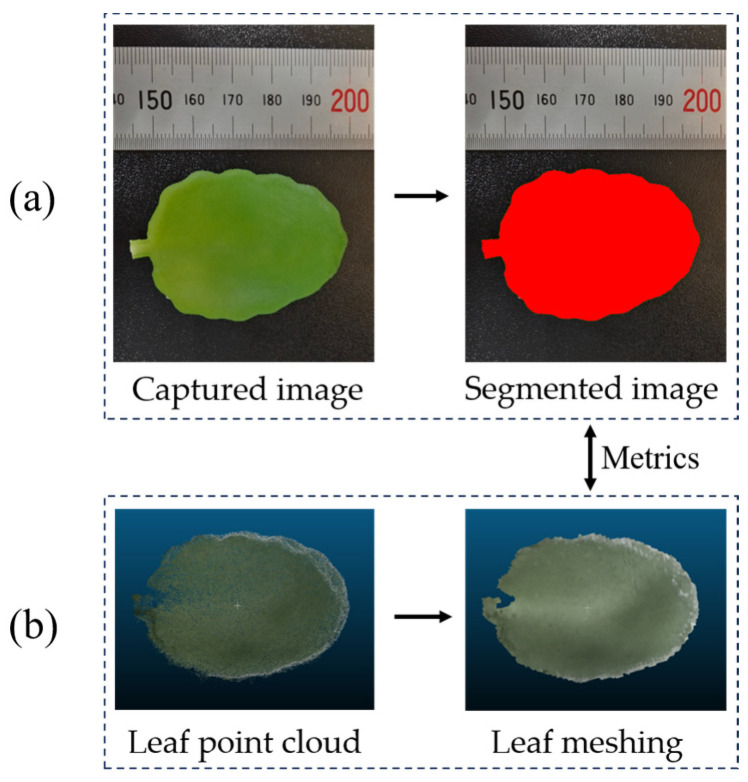
Example of individual leaf area calculation: (**a**) Leaf area measurement using ImageJ and (**b**) mesh reconstruction from a single leaf point cloud.

Leaf area and plant height are used to compare phenotypic parameters. As shown in Figure 9a, the ground truth leaf area was obtained by taking the average of three times of manual measurement using ImageJ software (version 1.54f). The reconstructed leaf point cloud was subjected to Poisson surface reconstruction, according to the method in Figure 8, and the filtered mesh area was used as the test leaf area to obtain the leaf mesh, as shown in Figure 9b.

In the plant height assessment, measurements were taken from the root of the main stem to the top of the plant [28]. The true plant height was obtained by averaging three measurements with a vernier caliper, while the reconstructed height was determined by measuring the vertical distance between the bottom and top of the reconstructed point cloud. The formula for calculating plant height can be expressed as follows:

(11) $$H=\max\left(Z_{plant}\right)-\min\left(Z_{plant}\right)$$

where $\max\left(Z_{plant}\right)$ and $\min\left(Z_{plant}\right)$ represent the highest and lowest points, respectively, in the reconstructed plant point cloud.

The error between the reconstructed model and the ground truth for both leaf area and plant height was evaluated using the coefficient of determination (R^2^) and the mean absolute percentage error (MAPE), which are defined as follows:

(12) $$R^{2}=1-\frac{\sum_{i=1}^{N}{\;\left(\;y_{i}-{\;\hat{y}}_{i}\right)}^{2}}{\sum_{i=1}^{N}\;{\left(\;y_{i}-\bar{y}\right)}^{2}}$$

(13) $$\mathrm{MAPE}=\frac{1}{N}\sum_{i=1}^{N}\left|\;\frac{y_{i}-{\;\hat{y}}_{i}}{y_{i}}\;\right|\times 100\%$$

where $y_{i}$ is the *i*-th ground truth value, ${\;\hat{y}}_{i}$ represents the corresponding *i*-th sample measured value, $\bar{y}$ is the mean of all ground truth values, and N is the total number of measured samples.

##### Evaluation of Runtime Performance

We evaluated the performance of different models, including the number of parameters, GPU memory usage, and the inference time per input sample. Each input sample consists of one reference depth map and multiple source images.

## 3. Results

### 3.1. Results of Depth Map Evaluation

Table 1 presents the depth image reconstruction results for different methods. Compared to other state-of-the-art methods, Edge_MVSFormer further optimizes the MAE_Depth for both valid pixels and edge regions, achieving the best reconstruction results. Additionally, the proportions of pixels with depth errors less than 2 mm and 4 mm have both increased.

**Figure 10:**
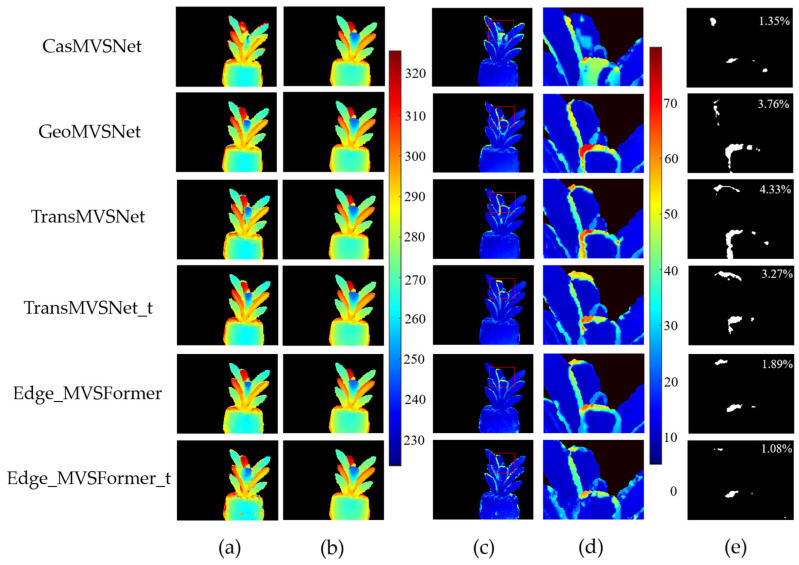
Depth test results for a specific viewpoint of a typical plant sample. (**a**) shows the test depth image filtered by valid pixel points, which correspond to regions in the original RGB image containing only the plant subject. (**b**) is the ground truth depth image, while (**c**) presents the absolute depth error between (**a**,**b**). (**d**) provides a magnified view of a 288 × 216 rectangular area in (**c**). (**e**) is a binary image highlighting regions in (**d**) where the depth error exceeds 40 mm. The percentage value indicates the proportion of white pixels (error > 40 mm) relative to the valid pixel points. The color bars represent an absolute depth in millimeters.

Figure 10 shows the depth images of typical test samples detected using different methods. As shown in Figure 10e, our method results in the smallest proportion of pixels with depth errors greater than 40 mm, indicating that the proposed algorithm significantly improves depth estimation, particularly for the depth estimation of leaf edges.

To evaluate the generalization ability of the network, we further collected multi-view images of the first ten plant scenes shown in Figure 2 under red and green lighting conditions and conducted evaluations on the depth images. The results in Table 2 indicate that our method achieves the best depth estimation performance under all three lighting conditions.

### 3.2. Results of Point Clouds Evaluation

Table 3 presents the evaluation results of the reconstructed point clouds obtained by different methods, including both the overall point clouds and the leaf edge point clouds extracted through segmentation. Compared to other state-of-the-art methods, Edge_MVSFormer shows slight improvements in both the mean absolute distance error (MAE_Distance) and overall performance (OP) for the overall point clouds, with a more significant improvement in the accuracy of the leaf edge point clouds. Specifically, compared to TransMVSNet_t, Edge_MVSFormer_t reduces MAE_Distance by 0.05 ± 0.02 mm and increases OP by 0.43% for the overall point clouds. In the edge region, MAE_Distance decreases by 0.13 ± 0.02 mm, and OP increases by 2.22%, further demonstrating the effectiveness of incorporating edge information and edge-aware loss.

### 3.3. Results of Phenotypic Parameters Evaluation

**Figure 11:**
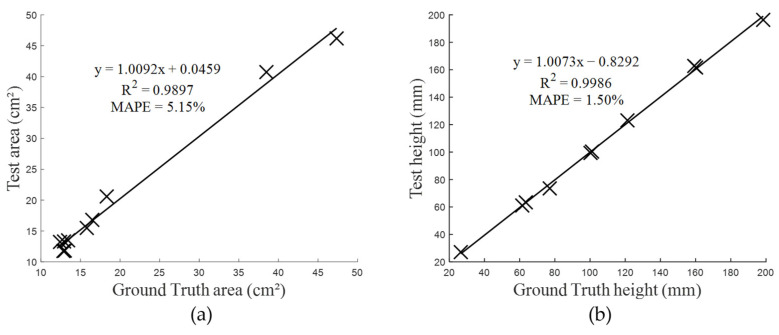
(**a**) Comparison between our measured and ground truth area results for 10 leaves. (**b**) Comparison between our measured and ground truth plant height results for 10 test model plants.

Figure 11 presents the evaluation results of the phenotypic parameters—leaf area and height. Panel (a) shows the leaf area calculation for 10 leaves, with a linear fit of y = 1.0092x + 0.0459, R^2^ = 0.9897, and MAPE = 5.15%. Panel (b) illustrates the calculated plant height for 10 plants, with a linear fit of y = 1.0073x − 0.8292, R^2^ = 0.9986, and MAPE = 1.50%. The results show a high degree of accuracy in the estimation of both leaf area and plant height, as evidenced by the high R^2^ values and relatively low MAPE.

### 3.4. Results of Runtime Performance Evaluation

Table 4 presents key metrics for the models CasMVSNet, GeoMVSNet, TransMVSNet, and the proposed Edge_MVSFormer, including the number of parameters, GPU memory usage, and inference time per sample. As the first lightweight cascaded network, CasMVSNet has fewer parameters and a shorter inference time, paving the way for lightweight MVS tasks. Due to its complex dual-branch structure, GeoMVSNet has a large number of parameters and relatively high memory consumption. TransMVSNet has 1.15 million parameters, 5513 MB of memory usage, and an inference time of 0.53 s. In contrast, Edge_MVSFormer strikes a good balance between model complexity and inference speed, making it a competitive choice for applications requiring both high accuracy and computational efficiency.

## 4. Discussion

In this study, a deep learning-based approach is proposed to generate high-quality 3D plant reconstructions from multi-view RGB images. Firstly, the multi-view RGB images and their corresponding edge information are used as network inputs, and the focal loss function and edge-aware loss function are introduced to enhance the accuracy of the generated depth map. The output is the depth image of a reference view, and then multi-view depth images are fused to generate a high-quality point cloud. The results show that adopting edge features in plant scenes can significantly improve the accuracy of 3D plant reconstruction, especially in the leaf edge region, as demonstrated in both the depth image and the point cloud evaluation. Specifically, compared to TransMVSNet_t, our Edge_MVSFormer_t achieved notable improvements in the reconstruction of depth images. The MAE_Depth in the depth image decreased, especially in the edge region; the percentage of pixels with depth errors less than 2 mm and 4 mm increased. In the depth image evaluation under three different lighting conditions, the depth estimation error was smallest under normal lighting, followed by green light, with the poorest performance under red light. This phenomenon is likely closely related to the performance of the depth estimation model, which depends on the distribution and quality of the training data. Specifically, the depth estimation model used in the experiments was trained on the DTU and BlendedMVS datasets. Although the DTU dataset includes various lighting conditions with different brightness levels, providing the model with a certain degree of generalization ability and allowing it to adapt to red and green lighting environments to some extent, the lack of targeted color lighting information in the training data leads to lower performance under these special lighting conditions compared to normal lighting. In the 3D point cloud evaluation, the MAE_Distance of the overall point cloud and the leaf edge point cloud was reduced to varying extents, and the OP increased by 0.43% and 2.22%, respectively. An increase in Acc but a slight decrease in Comp was also observed. The decrease in completeness can be attributed to the enhanced edge features improving the accuracy of the leaf edges in the depth image, thus reducing the number of inaccurate points in the generated point cloud. However, the distance to the generated point cloud for the ground truth points might be slightly increased, thus decreasing the Comp. It can also be noticed that the MAE_Distance of the 3D point cloud is much smaller than the MAE_Depth of the depth image, which is due to the fact that the process of fusing multiple depth maps into a point cloud selects 10 source images adjacent to the reference view for fusion and generates a point as long as 4 corresponding points in the source image satisfy the geometric consistency of the feature point in the reference view. Additionally, the Acc of the leaf edge point cloud is lower than that of the overall point cloud, but its Comp is higher, resulting in a higher OP compared to the overall point cloud. A potential factor influencing the evaluation results is the method used to obtain the ground-truth point cloud. This difference may arise from the fact that the ground-truth point cloud was acquired using the Freescan X3 scanner, which performs well in reconstructing leaf surfaces but struggles with inner stems in shaded areas. When generating the ground-truth point cloud, we adopted a multi-viewpoint cloud fusion strategy and used the ICP (Iterative Closest Point) algorithm to align and merge point clouds from different perspectives, which, to some extent, alleviates the sparsity issue in shaded areas. In the point cloud reconstructed from multi-view images, leaves from different directions also occlude inner stems in shaded regions, and these occlusion effects are similarly reflected in the reconstructed point cloud. Overall, since both the ground-truth point cloud and the reconstructed point cloud exhibit similar sparsity patterns, this sparsity is unlikely to have a significant impact on the Acc and Comp calculations of the overall point cloud. In the measurement of phenotypic parameters, the R^2^ and MAPE of leaf area correlation analysis were 0.9897 and 5.15%, respectively, and the R^2^ and MAPE of plant height regression analysis were 0.9986 and 1.50%, respectively. These results indicate that the proposed method for estimating phenotypic parameters is reliable and consistent across plant specimens and that the method is well suited for quantitative phenotyping in plant research and provides valuable insights for plant growth analysis and breeding applications.

TransMVSNet [23], a transformer-based MVS network, was selected as a benchmark for comparison. TransMVSNet has shown excellent performance on various 3D reconstruction datasets, such as DTU [15], Tanks and Temples [29], etc. One of its key innovations is the feature-matching transformer, which combines self-attention and cross-attention mechanisms to more effectively aggregate both intra- and inter-image contextual information, thereby improving feature-matching accuracy. However, achieving high-precision reconstruction of leaf edges remains particularly challenging in plant reconstruction. To address this issue, edge information was incorporated into the input during model training, and special emphasis was placed on the leaf edge regions in the loss function to improve overall 3D plant reconstruction accuracy.

Despite the promising results, this study still has certain limitations. First, due to the high time cost of data acquisition and preprocessing, only 20 model plants were collected for the experiment in this study—10 for network fine-tuning and 10 for testing. This limited sample size may affect the model’s ability to generalize across diverse plant types. Additionally, pre-training on a non-plant dataset followed by fine-tuning on a plant dataset may not fully mitigate the domain shift problem. Future research could explore more efficient domain adaptation strategies [30] to enhance the model’s adaptability and generalization to new environments. Second, point-to-point ICP [26] is widely used due to its computational efficiency and simplicity. However, it struggles with point clouds from the same implicit surface, as distance calculations depend on sampling density. In contrast, point-to-plane ICP [31] minimizes errors perpendicular to the surface, improving alignment accuracy in such cases. However, applying point-to-plane ICP to plant point clouds presents unique challenges, primarily due to the significant variation in surface curvature of plant leaves (such as veins and leaf edges), which makes consistent normal estimation difficult. This challenge is particularly pronounced for point-to-plane ICP, which relies on accurate normal estimation to minimize alignment errors, and the computational cost of normal estimation for plant point clouds is high. To balance accuracy with computational efficiency, this study opted for point-to-point ICP. Similarly, projection-based methods could improve the accuracy of point-to-surface perpendicular distance calculations when evaluating reconstructed point clouds. However, due to the high computational cost of precise normal estimation, this approach was not implemented but remains a promising direction for future research. A potential improvement could involve designing robust strategies for normal estimation tailored for plant structures, enabling precise surface alignment while maintaining computational feasibility. Additionally, hybrid approaches that integrate point-to-point and point-to-plane constraints could enhance point cloud alignment and evaluation without excessive computational overhead. Finally, this study did not specifically optimize the deep learning network architecture and hyperparameters, which may limit its performance. Future studies will focus on expanding data volume and diversity, improving preprocessing and augmentation techniques, and exploring multimodal data fusion. For example, deploying a novel LiDAR system on a mobile platform to capture real-world outdoor plant data could increase sample size and diversity, enhancing the model’s ability to generalize across different plant types and environments. Moreover, optimizing data preprocessing and applying data augmentation techniques could reduce the time cost associated with data acquisition and preprocessing. These improvements are expected to significantly advance 3D plant reconstruction, providing more effective tools for plant science and practical applications.

In conclusion, this study demonstrates a deep learning-based approach for high-quality 3D plant reconstruction from multi-view RGB images. By combining RGB images with edge information and introducing an edge-aware loss function, this study significantly improves the accuracy of leaf edge depth estimation, thereby enhancing overall 3D reconstruction quality. Experimental results confirm that the enhanced edge features greatly improve the accuracy of 3D plant reconstruction, especially in the leaf edge region of depth images and point clouds. The method in this paper not only achieves accurate 3D plant reconstruction with low computational cost but also yields highly accurate phenotypic parameter measurements. This study is expected to provide a more efficient and cost-effective solution for 3D plant reconstruction and phenotypic measurements, which will promote plant phenotyping research and facilitate the practical application of precision agriculture.

## Figures and Tables

**Table 1:** Depth error assessment of valid pixel points in reconstructed depth images.

Method	MAE_Depth (mm) ↓		EPE_2 ↑		EPE_4 ↑	
	Overall	Edge	Overall	Edge	Overall	Edge
CasMVSNet	7.37 ± 1.37	12.47 ± 1.95	36.97%	22.15%	53.68%	41.26%
GeoMVSNet	4.91 ± 0.79	8.71 ± 1.43	43.64%	33.19%	64.01%	53.46%
TransMVSNet	5.01 ± 0.81	9.18 ± 1.51	43.48%	28.13%	63.76%	52.37%
TransMVSNet_t	4.87 ± 0.78	8.74 ± 1.43	43.91%	28.67%	63.99%	52.89%
Edge_MVSFormer	4.50 ± 0.73	6.80 ± 1.06	44.54%	38.65%	65.44%	58.05%
Edge_MVSFormer_t	4.41 ± 0.71	6.54 ± 1.07	45.11%	39.89%	65.75%	59.32%

**Table 2:** Comparison of depth errors in depth images under different lighting conditions.

Model	Normal (mm)	Red (mm)	Green (mm)
CasMVSNet	7.34 ± 1.36	7.42 ± 1.47	7.44 ± 1.40
GeoMVSNet	5.04 ± 0.83	5.13 ± 0.81	5.08 ± 0.85
TransMVSNet	5.05 ± 0.82	5.23 ± 0.88	5.14 ± 0.84
Edge_MVSFormer	4.57 ± 0.73	4.76 ± 0.78	4.74 ± 0.81

**Table 3:** Performance assessment of the reconstructed point cloud.

Method	MAE_Distance (mm) ↓		Acc ↑		Comp ↑		OP ↑	
	Overall	Edge	Overall	Edge	Overall	Edge	Overall	Edge
CasMVSNet	1.36 ± 0.20	0.67 ± 0.11	38.27%	28.69%	44.69%	46.31%	41.48%	37.50%
GeoMVSNet	1.11 ± 0.17	0.52 ± 0.08	41.49%	39.36%	53.67%	58.17%	47.58%	48.77%
TransMVSNet	1.16 ± 0.17	0.54 ± 0.09	42.72%	35.08%	52.36%	57.55%	47.54%	46.32%
TransMVSNet_t	1.14 ± 0.18	0.55 ± 0.09	42.65%	35.61%	52.14%	57.96%	47.40%	46.79%
Edge_MVSFormer	1.08 ± 0.16	0.43 ± 0.07	43.23%	41.37%	50.83%	56.33%	47.70%	48.85%
Edge_MVSFormer_t	1.09 ± 0.16	0.42 ± 0.07	43.41%	42.83%	51.22%	55.18%	47.83%	49.01%

**Table 4:** Comparison of model parameters.

Model	Params (M)	GPU Mem (MB)	Inference Time (s)
CasMVSNet	0.93	5487	0.17
GeoMVSNet	15.31	7235	0.46
TransMVSNet	1.15	5513	0.53
Edge_MVSFormer	1.15	5572	0.54

## Data Availability

The dataset and code used in this study are publicly available at the webpage https://zenodo.org/records/15086606 with a DOI: 10.5281/zenodo.15086606, accessed on 19 March 2025.

## References

[1] Okura F. (2022). 3D modeling and reconstruction of plants and trees: A cross-cutting review across computer graphics, vision, and plant phenotyping. Breed. Sci..

[2] Kochi N., Hayashi A., Shinohara Y., Tanabata T., Kodama K., Isobe S. (2022). All-around 3D plant modeling system using multiple images and its composition. Breed. Sci..

[3] Phattaralerphong J., Sinoquet H.J.T.P. (2005). A method for 3D reconstruction of tree crown volume from photographs: Assessment with 3D-digitized plants. Tree Physiol..

[4] Paturkar A., Gupta G.S., Bailey D. (2020). Non-destructive and cost-effective 3D plant growth monitoring system in outdoor conditions. Multimed. Tools Appl..

[5] Ubbens J., Cieslak M., Prusinkiewicz P., Stavness I. (2018). The use of plant models in deep learning: An application to leaf counting in rosette plants. Plant Methods.

[6] Yang D., Yang H., Liu D., Wang X.J.C. (2024). Research on automatic 3D reconstruction of plant phenotype based on Multi-View images. Comput. Electron. Agric..

[7] He Y., Yu Z., Deng Y., Deng J., Cai R., Wang Z., Tu W., Zhong W. (2024). AHP-based welding position decision and optimization for angular distortion and weld collapse control in T-joint multipass GMAW. J. Manuf. Process..

[8] Andujar D., Calle M., Fernandez-Quintanilla C., Ribeiro A., Dorado J. (2018). Three-Dimensional Modeling of Weed Plants Using Low-Cost Photogrammetry. Sensors.

[9] Wang F., Zhu Q., Chang D., Gao Q., Han J., Zhang T., Hartley R., Pollefeys M. (2024). Learning-based Multi-View Stereo: A Survey. arXiv.

[10] Bi R., Gan S., Yuan X., Li R., Gao S., Yang M., Luo W., Hu L. (2023). Multi-View Analysis of High-Resolution Geomorphic Features in Complex Mountains Based on UAV–LiDAR and SfM–MVS: A Case Study of the Northern Pit Rim Structure of the Mountains of Lufeng, China. Appl. Sci..

[11] Sun S., Li C., Chee P.W., Paterson A.H., Meng C., Zhang J., Ma P., Robertson J.S., Adhikari J.J.C. (2021). High resolution 3D terrestrial LiDAR for cotton plant main stalk and node detection. Comput. Electron. Agric..

[12] Nguyen T.T., Slaughter D.C., Max N., Maloof J.N., Sinha N. (2015). Structured Light-Based 3D Reconstruction System for Plants. Sensors.

[13] Teng X., Zhou G., Wu Y., Huang C., Dong W., Xu S. (2021). Three-Dimensional Reconstruction Method of Rapeseed Plants in the Whole Growth Period Using RGB-D Camera. Sensors.

[14] Hu Y., Wang L., Xiang L., Wu Q., Jiang H. (2018). Automatic non-destructive growth measurement of leafy vegetables based on kinect. Sensors.

[15] Aanæs H., Jensen R.R., Vogiatzis G., Tola E., Dahl A.B. (2016). Large-Scale Data for Multiple-View Stereopsis. Int. J. Comput. Vis..

[16] Galliani S., Lasinger K., Schindler K. Massively parallel multiview stereopsis by surface normal diffusion. Proceedings of the 2015 IEEE International Conference on Computer Vision (ICCV).

[17] Schonberger J.L., Frahm J.M. Structure-from-motion revisited. Proceedings of the 2016 IEEE Conference on Computer Vision and Pattern Recognition (CVPR).

[18] Collins R.T. A space-sweep approach to true multi-image matching. Proceedings of the CVPR IEEE Computer Society Conference on Computer Vision and Pattern Recognition.

[19] Yao Y., Luo Z., Li S., Fang T., Quan L. Mvsnet: Depth inference for unstructured multi-view stereo. Proceedings of the European Conference on Computer Vision (ECCV).

[20] Gu X., Fan Z., Zhu S., Dai Z., Tan F., Tan P. Cascade cost volume for high-resolution multi-view stereo and stereo matching. Proceedings of the IEEE/CVF Conference on Computer Vision and Pattern Recognition.

[21] Zhang Z., Peng R., Hu Y., Wang R. Geomvsnet: Learning multi-view stereo with geometry perception. Proceedings of the IEEE/CVF Conference on Computer Vision and Pattern Recognition.

[22] Vaswani A., Shazeer N.M., Parmar N., Uszkoreit J., Jones L., Gomez A.N., Kaiser L. Attention is all you need. Proceedings of the Advances in Neural Information Processing Systems 30 (NIPS 2017).

[23] Ding Y., Yuan W., Zhu Q., Zhang H., Liu X., Wang Y., Liu X. Transmvsnet: Global context-aware multi-view stereo network with transformers. Proceedings of the IEEE/CVF Conference on Computer Vision and Pattern Recognition.

[24] Cao C., Ren X., Fu Y. (2022). MVSFormer: Multi-view stereo by learning robust image features and temperature-based depth. arXiv.

[25] Yao Y., Luo Z., Li S., Zhang J., Ren Y., Zhou L., Fang T., Quan L. Blendedmvs: A large-scale dataset for generalized multi-view stereo networks. Proceedings of the IEEE/CVF Conference on Computer Vision and Pattern Recognition.

[26] Xue S., Li G., Lv Q., Meng X., Tu X. (2019). Point Cloud Registration Method for Pipeline Workpieces Based On NDT and Improved ICP Algorithms. IOP Conf. Ser. Mater. Sci. Eng..

[27] Zhang J., Li S., Luo Z., Fang T., Yao Y. (2022). Vis-MVSNet: Visibility-Aware Multi-view Stereo Network. Int. J. Comput. Vis..

[28] Zhu X., Huang Z., Li B.J.P. (2024). Three-Dimensional Phenotyping Pipeline of Potted Plants Based on Neural Radiation Fields and Path Segmentation. Plants.

[29] Knapitsch A., Park J., Zhou Q.Y., Koltun V. (2017). Tanks and temples. ACM Trans. Graph..

[30] Ganin Y., Lempitsky V. Unsupervised domain adaptation by backpropagation. Proceedings of the International Conference on Machine Learning.

[31] Chen Y., Medioni G. (1992). Object modelling by registration of multiple range images. Image Vis. Comput..

